# The Relationship between Depressive Symptoms, Rumination, and Suicide Ideation in Patients with Depression

**DOI:** 10.3390/ijerph192114492

**Published:** 2022-11-04

**Authors:** Yi-Hsuan Chiang, Yu-Chin Ma, Yu-Chuan Lin, Jin-Ling Jiang, Mei-Hui Wu, Kuo-Cheng Chiang

**Affiliations:** 1 Department of Nursing, Fu Jen Catholic University, New Taipei City 24205, Taiwan; 2 Department of Nursing, Tzu Chi University, Hualien 97004, Taiwan; 3 Department of Nursing, Tzu Chi University of Science and Technology, Hualien 970302, Taiwan; 4 Psychiatric Ward for Hualien Tzu Chi Hospital, Hualien 97004, Taiwan

**Keywords:** depressive symptoms, rumination, suicide ideation

## Abstract

The relationship between suicide and rumination in depression is a recent topic of attention in mental health. The purpose of this study was to investigate the relationship between demographic variables, depressive symptoms, rumination, and suicide ideation in patients with depression, as well as the predictors of suicide ideation. Research design: A cross-sectional study of 95 subjects with depression recruited intentionally from the psychiatric ward of Tzu Chi Hospital. The questionnaire included demographic data, the Beck Depression Inventory-II, the Ruminative Response Scale, and the Beck Scale for Suicide Ideation. Independent sample *t*-test, Pearson product difference correlation, and the stepwise regression test were adopted for data analysis. Results: Age (r = −0.41, *p* < 0.01), age at diagnosis (r = −0.34, *p* < 0.01), and sleep duration (r = −0.25, *p* < 0.05) were negatively correlated with rumination–reflection. The depressive symptoms (r = 0.72, *p* < 0.01) were positively correlated with rumination, whereas rumination (r = 0.57, *p* < 0.01) and suicide ideation were positively correlated. Depressive symptoms and rumination could predict suicide ideation, and the effective explanatory power reached 60%. Conclusions: If the patient with depression was younger or the patient was diagnosed at a younger age, the depressive symptoms of the reflection subscale of rumination thinking and suicide ideation were more serious. Our results indicate that clinicians who care for patients with depression should be aware of rumination and its impact on suicide ideation, specifically in younger patients.

## 1. Introduction

Suicide is a global mental health and social issue with complex causes and negative impacts that extend to society as a whole. Therefore, suicide is an important healthcare issue [1]. There are more than 800,000 suicide deaths worldwide each year, accounting for 1.4% of global deaths. Suicide results from a combination of multiple risk factors, such as psychological, social, cultural, and personal losses. According to statistics released by the Ministry of Health and Welfare in Taiwan, in 2020, there were 3656 suicide deaths (2404 males and 1252 females), for a suicide mortality rate of 15.5 out of every 100,000 people. Among the major causes of death in Taiwan, suicide ranks 11th (12th before 2020), and the suicide death rate for males is twice that of females. Suicidal behavior is an important element in the diagnostic criteria for depression. A total of 43% to 50% of suicidal patients suffer from depression, and 46% of patients who committed suicide exhibited repeated suicidal behavior [2]. The risk factors for repeated suicide attempts include mainly females, lack of appropriate medical care, emotional or cognitive impairment, suicidal behavior in the past 12 months, experiencing life stressors and stressful events, having poor impulse control, and abusing alcohol [2,3]. Depression and suicide are highly correlated. In Taiwan, a study conducted on factors for, and the prediction of, depression and suicide (including ideation and behavior) were identified. In their study, depression, negative thoughts, gender, fear and anxiety, marital status, caregiver age and gender, hypertension, and education were the most predictive characteristics of depression [4]. Among the reported cases of suicide, 80% were individuals with depression [4]. An Australian study discusses the findings that hospitalized depressive patients also have a poor prognosis, a high rate of completed suicide, and high ongoing morbidity [5]. Therefore, suicide prevention should be the primary focus of clinicians/medical staff, especially for those with depression.

### 1.1. Suicide and Depression Symptoms

Aside from depression, emotional or cognitive impairment, suicidal ideation within the past 12 months, stressful events (including life stressors), poor impulse control, and a history of alcohol abuse were recognized as reasons associated with repeated suicidal behaviors [3]. In the suicide reporting system in Taiwan, the causes of suicide among Chinese people were mainly due to interpersonal affection, mental illness, and family stress, among which interpersonal affection and mental illness accounted for 50% of the causes of suicide [6].

Patients with depression and bipolar affective disorder are at a high risk of death by suicide (30 to 33.9%) [7]. Motives to commit suicide include the intention to give up everything when faced with life obstacles that feel as though they cannot be overcome, a sense of despair or a strong desire to end the suffering that is believed to be endless, and the belief that one’s death will ease the burden on others [8]. Some clinical psychological indicators, such as depression, hopelessness, and impulsivity, are good predictors of suicidal ideation [9]. For suicide (including ideation and behaviors), depression and negative thoughts, fear and anxiety, and day and night reversal are more prominent [4].

### 1.2. Rumination

Rumination refers to constantly thinking of an idea, situation, or event/choice.

Nolen-Hoeksema proposed the response style theory, which states that when individuals face depression, choosing rumination will delay and aggravate depression [10]. Since this theory was proposed, many studies have explored the issue of rumination as a cognitive susceptibility factor for negative emotions. A ruminative response style is defined as an individual’s continuous attention to his or her emotional symptoms including possible causes, and the related consequences of behaviors and thoughts. Ruminative responses lead to the deterioration and prolongation of depressive emotions and symptoms. The literature has shown that rumination reaction style is a trait-like cognitive feature that can affect the cognitive and the cognition and behavior of individuals in response to negative emotions or stressful events [11]. The negative component of rumination is considered to be a coping mechanism for depression-related maladaptation [12].

### 1.3. The Relationships of Depressive Symptoms and Rumination with Suicidal Ideation

Recently, the relationship between suicidal ideation with suicide attempts and rumination has become a topic of concern to researchers. Some studies have suggested that rumination is significantly correlated with suicidal ideation in clinical practice and the community [13]. Treynor et al. divided rumination into (1) reflection (facing and solving problems and difficulties), and (2) brooding (discouraged and frustrated). The brooding style and reflection style of rumination had an impact on suicide attempts [14]. Rumination can predict suicide [15]. Because the persistence of negative thoughts allows patients to continue suicidal ideation, rumination is a good predictor of existing and persistent suicidal ideation [16]. Rumination correlated with suicidal ideation in the presence of depressive symptoms [17].

Despite the proposed relationship between rumination and depressive symptoms in the literature, there is a lack of research in Taiwan on this topic. The purpose of our study was to investigate the relationships and predictive factors associated with depression, rumination, and suicidal ideation among people in Taiwan.

## 2. Methods

### 2.1. Research Design and Participant

For this cross-sectional study, we collected the basic demographic data on 95 subjects which included gender, age (including age at diagnosis), marital status, education level, religion, and sleep duration. We included patients between 20 and 65 years of age with a confirmed diagnosis of depression, stable symptoms who were treated in an inpatient unit of a psychiatric ward, those who agreed to be interviewed and completed the questionnaire; we excluded patients with intellectual disturbances, schizophrenia, and alcohol or drug abuse.

### 2.2. Measures

#### 2.2.1. Basic Demographic

Gender, age, age at diagnosis, marital status, education level, religion, and sleep duration were collected for each participant.

#### 2.2.2. Ruminant Response Scale (RRS-C)

The Chinese version of this scale was developed by Huang (2015) who recruited 143 subjects diagnosed with depression-related diseases from psychiatric clinics wards in Taiwan. This assessment tool included 22 questions. The Chinese version of the Ruminant Response Scale uses responses ranging from one point, which represents the respondent never thinking about the issue, to four points, which represents the respondent always thinking about the item; the two factors, brooding, and reflection, for the RRS-C. Cronbach’s alpha ranges from 0.71 to 0.77 [18].

#### 2.2.3. Beck Depression Inventory-II (BDI-II)

The Chinese version of the Beck Depression Inventory was published in 1996, it was translated into Chinese by Chen in 2000, becoming the first Chinese version of the BDI-II. There are a total of 21 items on this scale. The Cronbach’s alpha of the overall scale is 0.94, and the half-reliability of the scale is 0.91 [19].

#### 2.2.4. Beck Scale for Suicidal Ideation (BSS)

The BSS contains 21 questions that aim to measure suicidal thoughts, attitudes, and plans. The first 5 questions screen the respondent’s attitudes toward life and death. If the assessment indicates active or passive suicide ideation, questions 6–20 are then to be answered. The Cronbach’s alpha is 0.87~0.90. The test-retest reliability is 0.54 [20].

### 2.3. Research Ethics

This study was approved by the Human Research Committee of Tzu Chi Hospital (IRB 109-006-B). The investigator personally explained the key points of the study and its significance to potential participants and fully explained to these individuals their rights and why the study may be of interest to them. Participants were enrolled after signing a consent form. The participants were enrolled from September 2020 to December 2021 at the psychiatric ward of Tzu Chi Hospital in Taiwan.

### 2.4. Data Analysis

The collected questionnaires were preliminarily processed and the data were statistically analyzed using SPSS 22/Windows. The statistical methods were as follows. Basic characteristics were presented as the mean, standard deviation, median, and percentage of the number of people. The Pearson correlation test was used to determine the relationship between basic demographic and rumination and suicidal ideation. Stepwise regression was used to predict suicidal ideation by age, age at diagnosis, sleep duration, depressive symptoms, and rumination.

## 3. Results

### 3.1. Basic Demographic

As seen in Table 1, there were 95 subjects in this study; the average age was 49.54 years (SD: 14.03). The majority were female (60; 63%), and 43% were married. A high school education (76%) was the most prominent education level among the participants. The religious believers were mostly Eastern (76, 80%). The average age at diagnosis was 37.92 years, and the average nighttime sleep duration while hospitalized was 458 min. The average rumination total score for depression patients was 36.71 (SD: 12.74), and the two factors for brooding, and reflection were 7.6 (SD: 2.86) and 4.08 (SD: 2.60), respectively. The mean depressive symptoms score was 25.59 (SD: 14.02), and the mean suicidal ideation score was 11.93 (SD: 11.44).

### 3.2. Correlations of Demographic, Depressive Symptoms, Rumination, with Suicidal Ideation in Patients with Depression

As seen in Table 2, the age of patients with depression was significantly negatively correlated with depressive symptoms (t = −0.44, *p* < 0.01) and suicidal ideation (t = −0.55, *p* < 0.01). The younger the patient, the more severe the depressive symptoms and suicidal ideation. The age of depressed patients was significantly negatively correlated with the reflection factor of rumination (t = −0.41, *p* < 0.01), indicating that the younger the patient, the more serious. The age at diagnosis for a depressed patient was significantly negatively correlated with depressive symptoms (t = −0.32, *p* < 0.01) and suicidal ideation (t = −0.41, *p* < 0.01). The patient’s age at depression diagnosis was significantly negatively correlated with the reflection factor of rumination (t = −0.34, *p* < 0.01), indicating that the younger the age at diagnosis, the more severe the reflection factor of rumination. Sleep duration was significantly negatively correlated with the reflection factor (t = −0.25, *p* < 0.05), indicating that the reflection factor of rumination was more severe when the sleep duration was shorter. Depression symptoms were positively correlated with the total rumination score (t = 0.72, *p* < 0.01), brooding factor score (t = 0.59, *p* < 0.01), and reflection factor score (t = 0.71, *p* < 0.01). Suicidal ideation was positively correlated with the total rumination score (t = 0.57, *p* < 0.01), brooding factor score (t = 0.42, *p* < 0.01), and reflection factor score (t = 0.61, *p* < 0.01). The results suggest that the more serious the rumination was, the more serious the suicidal ideation.

### 3.3. Predictive Factors for Suicidal Ideation in Patients with Depression

Table 3 shows the results of the stepwise regression analysis. The three models effectively explained 60% of suicidal ideation (F (4, 94) = 33.830, *p* < 0.001). When controlling for age and age at diagnosis, the explanatory power of depressive symptoms and rumination was significant (depressive symptoms: β = 0.395 (t = 3.67, *p* < 0.001); rumination: β = 0.225 (t = 2.29, *p* < 0.05), indicating that the higher the depressive symptoms and rumination scores, the higher the suicidal ideation score was, in other words, the serious depressive symptoms, the stronger the suicidal ideation were, and the more severe the rumination, the stronger the suicidal ideation was.

## 4. Discussion

The results of this study indicated that age, age at diagnosis, and sleep pattern were negatively correlated with the reflection factor of rumination and that depression symptoms, rumination, and suicidal ideation were positively correlated. Age and age at diagnosis were negatively correlated with the reflection factor score of rumination, suggesting that the younger the age (including age at depression diagnosis) the more severe rumination. Our finding is similar to previous studies [21]. In addition, studies have suggested that rumination in depressed patients will decrease over time during different life cycles. However, suicidal attempts increase with aging [22]. One reason that older age and older age at diagnosis result in less rumination may be that older people have a better ability to regulate their emotions, leading to less rumination than that observed in young people.

Sleep duration was negatively correlated with the reflection factor of rumination, suggesting that depressed patients with less nighttime sleep duration exhibited more severe rumination. This result is consistent with that reported by Rogers et al. (2017), who studied 492 discharged psychosis patients. A previous study of sleep and rumination showed that there was a significant positive correlation between insomnia and rumination (Rogers et al., 2017), with similar results reported by Carney et al. (2013). Carney et al. noted that rumination was correlated with poor sleep quality; in their study of 66 patients with clinical depression and insomnia, rumination was correlated with sleep quality [23]. Sleep hygiene and depression are mutually causal, and sleep status is also an important indicator for assessing the physical and mental conditions of patients. The core feature of depression is long-term and difficult-to-control negative emotions resulting in a cycle of sadness and negative thinking [18]. Therefore, there must be a correlation between sleep and rumination.

Age and sleep duration were negatively correlated with the reflection factor of rumination. For young depressed patients with poor sleep hygiene or quality, the reflection factor of rumination becomes more serious, especially with regard to self-anger and self-isolation (for example, “I want to be angry with myself”, “I listen to sad music”, “I isolate myself and think about the reasons why I feel sad”). In this study, the average age of the participants was 49 years and the average age at diagnosis was 37 years. Due to the long-term nature of depression, professionals often provide counseling opportunities during inpatient treatment so that patients feel like they are not alone in facing the disease and have the opportunity to learn disease-related knowledge and how to regulate their emotions. However, younger patients, especially those who are hospitalized for the first time and have fewer hours of sleep, coupled with severe symptoms of depression, face emotional distress or depression symptoms on their own and are more likely to adopt self-imposed isolation or to be angry with themselves. The age at diagnosis and depression levels are factors in the assessment of suicide or rumination.

In this study, there was a positive correlation between rumination and suicidal ideation, indicating that a higher rumination score on admission may signify more serious suicidal ideation. A previous study found that correlations between depression symptoms and rumination and between suicidal thoughts that persisted over a long period and rumination were similar [14]. Depressive symptoms and rumination can both predict suicidal ideation. That study also reported similar correlations for long-term depressive symptoms and rumination and suicidal thoughts [15]. The results from this study will help to better understand the relationship between rumination and suicidal ideation in depressed patients. In addition, we also further confirmed the important influence of rumination on depression; that is, rumination affects both depressive symptoms and suicidal ideation. Therefore, when assessing suicidal ideation in patients with clinical depression, rumination should be included as one of the assessment criteria. Furthermore, from a cultural perspective, this study focused on patients with depression in the context of Chinese culture. In Chinese culture, mental illness is regarded as taboo, and repressed feelings or emotions are common. Therefore, negative thoughts, such as self-blame and hopelessness, often cannot be shared with others. As such, future studies should re-examine other factors that affect rumination with regard to depression in individuals with Taiwanese cultural characteristics, to increase the applicability of the results of this study.

## 5. Conclusions

Our study further proved rumination as a predictive factor for suicidal ideation, provided a supplementary perspective on rumination, and clarified the relationship between rumination and suicidal ideation. In addition, in clinical practice, the findings herein will help medical teams address the negative effects of rumination on patients. When caring for depressed patients, especially those who are younger and have poor sleep hygiene, medical staff should pay special attention to their rumination and suicidal thoughts. In young depressed patients, negative thoughts can be effectively mitigated by reducing feelings of self-blame and self-isolation through talking and activities, and by caring about why a patient is angry with him or herself, thereby focusing on the self-focus aspect of rumination. Rumination is related to suicidal ideation. Therefore, suicide assessments and rumination questionnaires should be administered at the admission of depression patients with suicidal thoughts, to comprehensively assess the risk and prevention of suicide.

Our study had a few limitations which included the cross-sectional design, single-region data, and a single point of time for analysis. A longitudinal study on rumination in depressed patients should be designed in the future to analyze the temporal trends and differences in depression levels as factors in the assessment of suicide or rumination.

## Figures and Tables

**Table 1:** Demographic and key variables of participants (*N* = 95).

Variables	*N*	%	Mean	SD
Gender				
male	35	37		
female	60	63		
Religion				
eastern	76	80		
western	19	20		
Education status				
secondary	22	24		
high School	73	76		
Married status				
no	54	57		
yes	41	43		
Age			49.54	14.03
Age at diagnosis			37.92	13.78
Sleep duration(min)			458.64	61.95
Depression			25.59	14.02
Suicide			11.93	11.44
Rumination			36.71	12.74
brooding			7.60	2.86
reflection			4.08	2.60

**Table 2:** The correlation of rumination and suicide (*N* = 95).

Variables	Depression	Rumination			Suicide
			Brooding	Reflection	
Age	−0.445 **	−0.199	−0.167	−0.413 **	−0.559 **
Age at diagnosis	−0.320 **	−0.138	−0.037	−0.344 **	−0.415 **
Sleep duration	−0.113	0.152	−0.051	−0.257 *	−0.046
Depression		0.722 **	0.594 **	0.714 **	0.709 **
Suicide		0.577 **	0.426 **	0.610 **	

* *p* < 0.05, ** *p* < 0.01.

**Table 3:** Analysis of predictors of suicidal ideation in patients with depression ( *N* = 95).

	Model 1		Model 2		Model 3	
	β	SE	β	SE	β	SE
Age	−0.513 ***	0.096	−0.271**	0.080	−0.302 **	0.079
Age at diagnosis	−0.068	0.097	−0.048	0.077	−0.053	0.075
Depression			0.573 ***	0.062	0.395 ***	0.088
Rumination					0.225 *	0.088
ΔR^2^	0.315		0.577		0.601	
*F*	21.121		41.431		33.830	

Dependent: suicide ideation. *** *p* < 0.001, ** *p* < 0.01, * *p* < 0.05.

## Data Availability

Not applicable.
